# A Tethered Bilayer Assembled on Top of Immobilized Calmodulin to Mimic Cellular Compartmentalization

**DOI:** 10.1371/journal.pone.0019101

**Published:** 2011-04-20

**Authors:** Claire Rossi, Samah Doumiati, Clarine Lazzarelli, Marilyne Davi, Fetta Meddar, Daniel Ladant, Joël Chopineau

**Affiliations:** 1 UMR 6022 CNRS, Université de Technologie de Compiègne, Compiègne, France; 2 Université de Nîmes, Nîmes, France; 3 Institut Pasteur, Unité de Biochimie des Interactions Macromoléculaires, CNRS URA 2185, Paris, France; 4 Institut Charles Gerhardt Montpellier, UMR 5253 CNRS-ENSCM UM2-UM1 Ecole Nationale Supérieure de Chimie, Montpellier, France; George Mason University, United States of America

## Abstract

**Background:**

Biomimetic membrane models tethered on solid supports are important tools for membrane protein biochemistry and biotechnology. The supported membrane systems described up to now are composed of a lipid bilayer tethered or not to a surface separating two compartments: a ”*trans*” side, one to a few nanometer thick, located between the supporting surface and the membrane; and a “*cis*” side, above the synthetic membrane, exposed to the bulk medium. We describe here a novel biomimetic design composed of a tethered bilayer membrane that is assembled over a surface derivatized with a specific intracellular protein marker. This multilayered biomimetic assembly exhibits the fundamental characteristics of an authentic biological membrane in creating a continuous yet fluid phospholipidic barrier between two distinct compartments: a “*cis*” side corresponding to the extracellular milieu and a “*trans*” side marked by a key cytosolic signaling protein, calmodulin.

**Methodology/Principal Findings:**

We established and validated the experimental conditions to construct a multilayered structure consisting in a planar tethered bilayer assembled over a surface derivatized with calmodulin. We demonstrated the following: *(i)* the grafted calmodulin molecules (in *trans* side) were fully functional in binding and activating a calmodulin-dependent enzyme, the adenylate cyclase from *Bordetella pertussis;* and *(ii)* the assembled bilayer formed a continuous, protein-impermeable boundary that fully separated the underlying calmodulin (*trans* side) from the above medium (*cis* side).

**Conclusions:**

The simplicity and robustness of the tethered bilayer structure described here should facilitate the elaboration of biomimetic membrane models incorporating membrane embedded proteins and key cytoplasmic constituents. Such biomimetic structures will also be an attractive tool to study translocation across biological membranes of proteins or other macromolecules.

## Introduction

Biomimetic membrane systems have been developed to study, in controlled conditions, the biological events occurring at the cell membrane interface.[1], [2] Over the past 25 years, biomimetic models have been continuously improved with the aim of better mimicking the natural environment of biological membranes while allowing deeper investigations of membrane processes with various surface sensitive techniques such as Surface Plasmon Resonance (SPR), Atomic Force Microscopy, Quartz Crystal Microbalance, neutron-reflectometry, etc…[3], [4] Introduction of tethered supported bilayers (or tethered bilayer membranes, *t*BLM) has been a major implementation toward the reconstitution of integral membrane proteins within a fluid hydrophobic environment that preserves their functional properties.[5], [6] These supported lipid bilayers have been widely used to characterize interaction of ligands with membranes, dynamics of membrane proteins[7] or even more complex receptor/ligand mediated intercellular contacts.[8], [9] Yet, in most cases, these reconstituted assemblies involved only the extracellular domains of cell receptors that were attached to the membrane bilayer in a manner preserving their lateral mobility[10], [11], but did not take into account the underlying cytosolic compartment.

**Figure 1:**
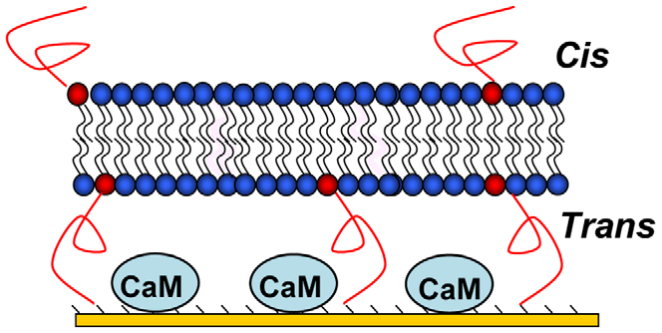
Scheme of the tethered bilayer formed over CaM molecules immobilized on top of an amine-coated surface.

Here, we present an improved model of biomimetic *t*BLM mimicking the three-dimensional architecture of a genuine biological membrane in that it defines a physical boundary between two distinct compartments, i.e. *cis* (or external) and *trans* (internal) sides (Figure 1). This was achieved by assembling a continuous tethered bilayer over a surface derivatized with the protein calmodulin (CaM) to serve as a specific cytoplasmic marker. CaM is a ubiquitous, highly conserved intracellular Ca^2+^ sensor, capable of binding and regulating diverse intracellular targets such as protein kinases, protein phosphatases, phosphodiesterases, and ion channels.[12] We established and validated the experimental conditions to assemble such a multilayered structure that preserves the functional activity of CaM and ensures the formation of a continuous yet fluid lipid bilayer acting as a protein-impermeable barrier between two distinct compartments. The simple and robust procedure elaborated here to assemble multilayered biomimetic structures will be instrumental to reconstitute multimolecular complexes involving both cytosolic and membrane embedded proteins such as those implicated in many cell signaling pathways and will also be useful to characterize protein translocation across membranes.

## Results and Discussion

**Figure 2:**
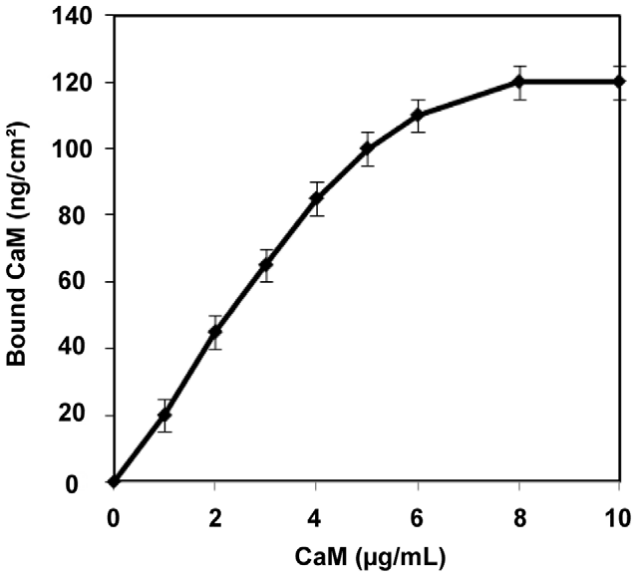
CaM immobilization on cysteamine gold surfaces. Amount of immobilized CaM per surface (ng/cm^2^) as a function of the concentration of the CaM solution (µg/mL) injected over the amine-coated gold surface. The protein surface coverage was calculated from SPR measurements; the averages and standard deviations were calculated from 8 independent measurements.

The first step of the surface construction was to immobilize CaM in a functional state between the substrate and the lipid bilayer (Figure 1). Amine coated gold surfaces were prepared by self-assembly of 2-aminoethanethiol on gold surfaces.[13] CaM was covalently coupled to the amino-grafted surface using EDC activation in the presence of calcium (2 mM) to stabilize its active conformation.[12] The optical thickness of the bound CaM layer was determined by SPR spectroscopy. The amount of immobilized CaM increased linearly with the concentration of CaM added onto the surface up to a saturation limit of 120 ng/cm^2^ which corresponds to about 40 000 molecules per µm^2^ (Figure 2).

In the second step, a biomimetic lipid membrane (*t*BLM) was assembled over the CaM layer. We have previously developed a one-step construction of a tethered lipid bilayer membrane (*t*BLM) based on EggPC/DSPE-PEG_3400_-NHS mixed vesicle deposition onto an amine-grafted surface in which the lipopolymers serve to covalently anchor the bilayer to the surface.[13], [14] We adapted this procedure to construct a tethered bilayer on the amine-coated surface derivatized with CaM. An EggPC vesicle suspension, doped with 5% (w/w ratio) DSPE-PEG_3400_-NHS, was deposited onto the CaM/amine-coated surface and allowed to react for 1 hr. The chip was then washed extensively in order to favor the fusion of the attached vesicles into a continuous planar bilayer and to remove the excess of unlinked vesicles. [13], [14]

**Figure 3:**
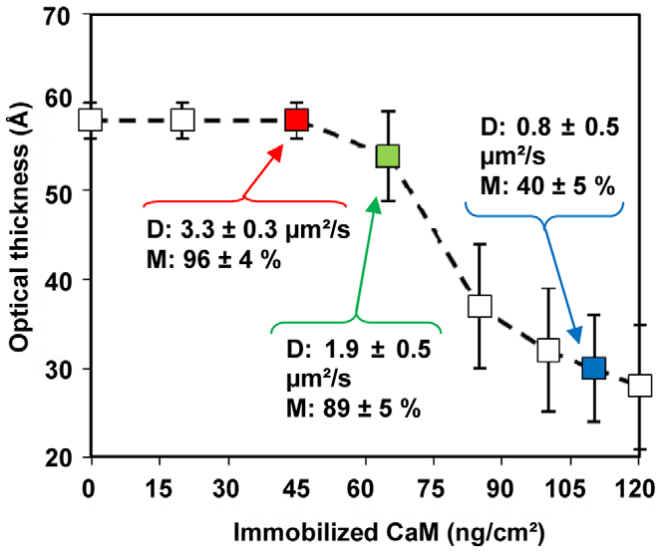
Characterization of the lipid architecture as a function of the CaM coverage. The optical thickness of the lipid deposit was monitored by SPR. Fluorescence measurements were performed to determine the lipid diffusion coefficient (D) and the lipid mobile fraction (M). The standard deviations of optical thickness measurements from 8 independent experiments are shown.

**Figure 4:**
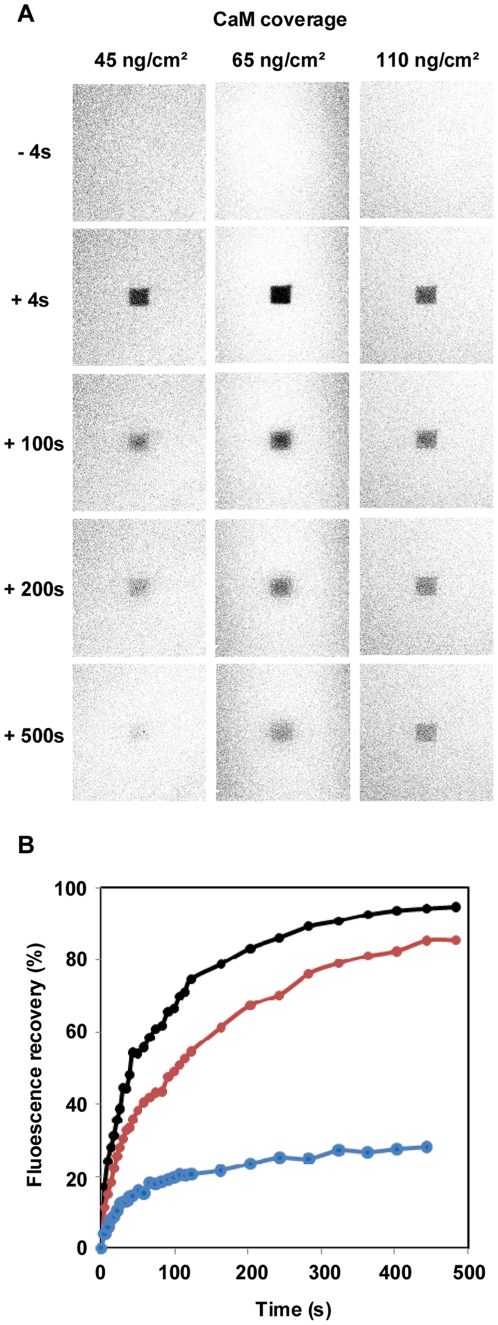
Evaluation of the lipid assembly fluidity. (A) Fluorescence photobleaching images as a function of time for lipid assemblies formed onto cysteamine-coated surface with different CaM coverages (45, 65 and 110 ng/cm^2^). (B) Fluorescence recovery curves after photobleaching of tethered lipid assemblies built on the surface with different CaM surface coverage: 45, 65 and 110 ng/cm^2^.

The properties of the covalently-bound lipid assembly, i.e. optical thickness and lipid fluidity (lateral diffusion coefficient and mobility of the fluorescent lipid DPPE-NBD, 2% mol), were characterized by SPR and by fluorescence recovery after photo-bleaching (FRAP) respectively. Figures 3 and 4 (and Table 1) show the characteristics of the assembled lipid structures as a function of the density of immobilized CaM. When the CaM coverage densities were below 45±5 ng/cm^2^, the optical thickness (58±2 Å) as well as the mobility of the fluorescent lipid probes of the tethered membrane (more than 95% of probes were mobile with a lateral diffusion coefficient of 3.3±0.3 µm^2^/s), were highly similar to that of bilayer membranes formed in the absence of CaM (Figure 3) and in good agreement with our previous studies.[13], [14] For higher CaM surface density, the optical thickness of the membrane was found to decrease significantly and also became highly variable from one experiment to another (see the standard deviation in Figure 3) likely as a result of the heterogeneity of the lipid deposit. The mobility of the fluorescent lipid probes decreased similarly. As reported in Table 1 and Figures 3 and 4, when the membrane was tethered on a surface covered with 100 ng/cm^2^ CaM, less than 40% of the fluorescent lipid probes were mobile with a diffusion coefficient below 1 µm^2^/s. Taken together, these SPR and FRAP measurements were in accordance with the formation of a continuous tethered bilayer on top of the immobilized CaM, when the protein density was below 45±5 ng/cm^2^ (i.e. lower than 15,000 molecules per µm^2^). In these conditions, assuming that CaM formed a single layer, the protein should cover less than 25% of the total surface. At higher CaM density, the coupling of the vesicles was reduced, likely due to steric constraints, and this precluded the assembly of a continuous planar bilayer.

**Table 1:** Tethered membrane fluidity.

Immobilized CaM *(ng/cm^2^)*	Diffusion coefficient *(*µ*m^2^s^−1^)*	Mobile fraction *(%)*
45	3.3±0.3	96±4
65	1.9±0.5	89±5
110	0.8±0.5	40±5

The lipid diffusion coefficients and the mobile fractions of the biomimetic constructions are given as a function of the initial CaM coverage. They are calculated from six independent measurements.

One critical issue to create an authentic boundary between two truly separated compartments is whether the bilayer was tightly sealed to fully insulate the underlying CaM molecules (*trans* side) from the bulk medium (*cis* side). To validate the barrier properties of the biomimetic assembly, we resorted to a CaM-dependent enzyme, the adenylate cyclase (AC) from *Bordetella pertussis*. This enzyme is a toxin able to invade eukaryotic target cells where it is activated more than 1000 fold upon binding to CaM to produce supraphysiologic levels of cAMP.[15] AC binds with a high affinity to CaM in solution and was also shown earlier to interact efficiently with CaM immobilized on plastic surface.[16]

**Figure 5:**
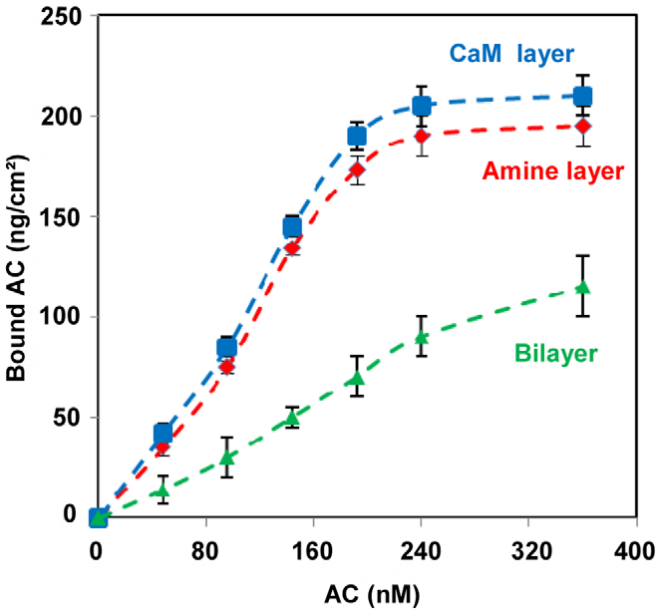
SPR quantification of AC binding to the different type of surfaces. AC binding to the different type of surfaces: cysteamine monolayer (red curve); cysteamine monolayer partially derivatized with CaM at a surface coverage of 45±5 ng/cm^2^ (blue curve); and tethered bilayer assembled over the CaM coated surface (at 45±5 ng/cm^2^; green curve). The amounts of AC bound to the different surfaces (in ng/cm^2^) as a function of the AC concentrations injected onto the surfaces, were determined by SPR spectroscopy. The average and standard deviation values were calculated from three independent measurements.

We first attempted to monitor the specific AC binding to the immobilized CaM by SPR spectroscopy (Figure 5). For this, the quantities of AC bound to the surfaces were evaluated from SPR measurements at the different steps of the tethered bilayer construction: firstly on the cysteamine monolayer, then on the partially covered CaM surface layer (45±5 ng/cm^2^) which still exposed a large area of free cysteamine, and finally over the tethered bilayer assembled over the immobilized CaM layer (45±5 ng/cm^2^). As shown in Figure 5 (red curve), up to 200 ng/cm^2^ of AC were able to bind to the cysteamine-coated surface in a non-specific manner. Indeed, proteins are known to adsorb non-specifically to amine-coated surface.[17]AC binding to the surface derivatized with CaM (blue curve) was not significantly different than that found with the cysteamine-coated surface. This was not surprising given that, at the CaM-coupling density used here (45±5 ng/cm^2^), a large area (about 75% of total surface) of free cysteamine layer was still accessible to the bulk medium. These results thus indicated that SPR could not be used to discriminate between the specific association of AC with the immobilized CaM and the non-specific adsorption of AC to the cysteamine-layer. AC was also found to adsorb to the lipid leaflet of the tethered bilayer although to a significantly lower level (green curve).

**Figure 6:**
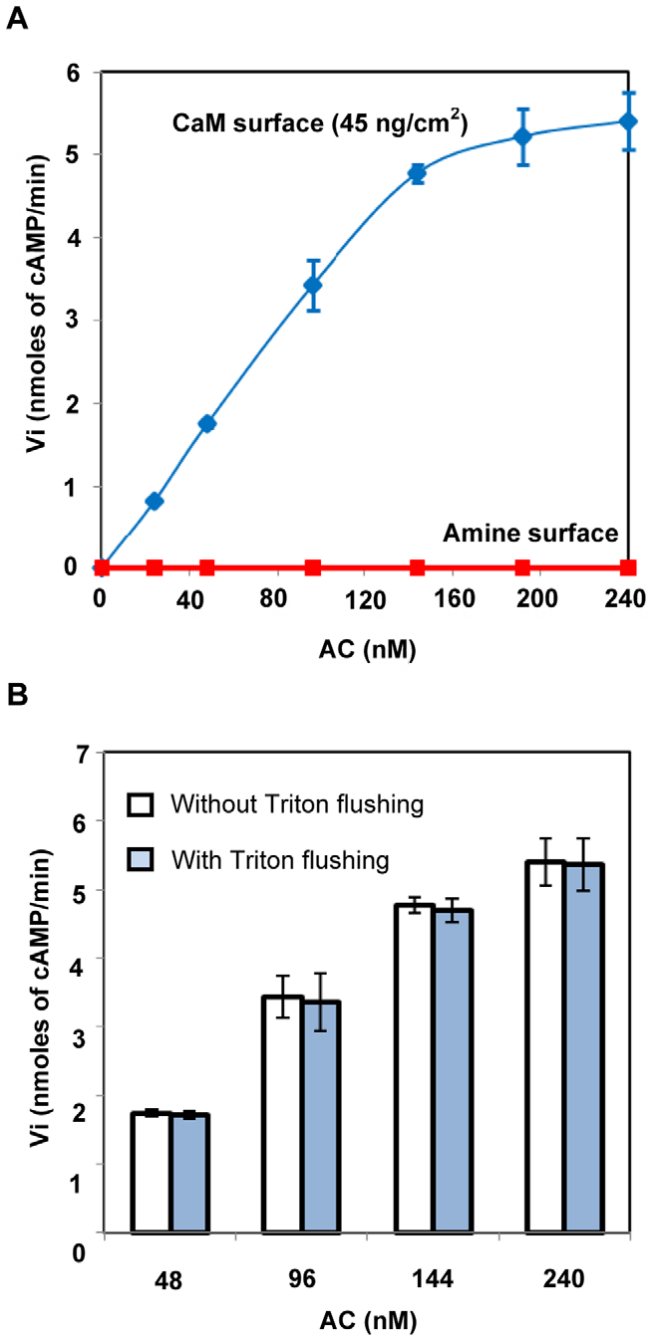
AC activation by the immobilized CaM. (A) Initial rates of AC activity (v_i_ in nmoles of cAMP/min) as a function of AC concentration (nM) in the solution deposited (or injected) on the indicated surfaces: cysteamine coated surface (red curve) and cysteamine-coated surface bearing 45±5 ng/cm^2^ of immobilized CaM (blue curve). The data and standard deviations were from at least six measurements with independent surfaces. (B) AC binding to the immobilized CaM was not impaired by the membrane solubilization procedure. Initial rates of AC activity (v_i_ in nmoles of cAMP/min) as a function of AC concentration in the solution (nM) deposited (or injected) onto the calmodulin coated amino-surfaces (45±5 ng CaM/cm^2^), were quantified either after washing of the surface with 5 mL of HBS buffer (white bars) or after washing of the surface with 2 mL of 0.1% Triton X-100 in HBS buffer followed by 5 mL of HBS buffer (blue bars). The enzymatic reaction medium (600 µL) was deposited onto the surface after removal of the HBS buffer and kinetics of Pi production were determined as mentioned above. The average and the standard deviations are from three measurements on independent surfaces.

**Figure 7:**
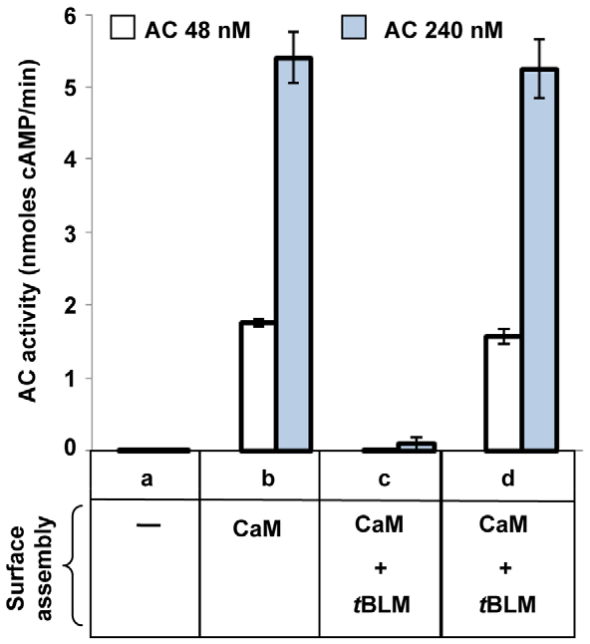
AC binding to the CaM/tethered lipid bilayer structures. The different surface constructions (in a, amine-coated surface alone; in b, CaM-coated surface at a density of 45±5 ng/cm^2^, in c and d, CaM-coated surface (at 45±5 ng/cm^2^) overlaid with a continuous tethered bilayer membrane, *t*BLM) were incubated with AC (48 nM or 240 nM) in HBS buffer for 30 min, and extensively washed first with HBS buffer, then with 0.1% Triton X-100 and finally with HBS buffer again. In d, the *t*BLM was disrupted by washing with 0.1% Triton X-100 before incubation with AC. AC binding to different surface constructions was monitored by measuring its enzymatic activity (expressed as initial rate of cAMP formation) with a colorimetric assay.[19] The results shown are the mean from 6 independent experiments.

To selectively quantify the enzyme bound to the CaM immobilized on the cysteamine surface, we exploited the fact that AC enzymatic activity is strongly stimulated (about a 1000 fold) upon association with its activator.[15] Furthermore, the CaM-activated AC has a high specific activity[18] that can be easily monitored *in vitro* with a colorimetric assay that was recently developed: AC converts ATP into cAMP and pyrophosphate. The latter can be further hydrolyzed by an exogenously added pyrophosphatase into two phosphate molecules that are quantitatively measured with a standard phosphomolybdate/malachite green assay.[19] As shown in Figures 6A and 7, the AC bound to the CaM-coated cysteamine surface (surface coverage of 45±5 ng/cm^2^) displayed a high enzymatic activity indicating that the immobilized CaM was functional in stimulating AC catalysis. In contrast, the AC non-specifically adsorbed on the cysteamine monolayer in the absence of CaM (as revealed by SPR measurements, see above Figure 5) did not exhibit any detectable enzymatic activity. Hence the enzymatic activity appeared as a highly selective reporter of the functional association of AC molecules with the immobilized CaM. In addition, we checked (Figure 6B) that the AC molecules were tightly associated to the attached CaM, and in particular, were not dissociated from the immobilized CaM upon extensive washing in the presence of non-ionic detergent (0.1% Triton X-100). This property was important as this detergent solution was used to solubilize the tethered bilayer as described in the following section.[20]

To probe the barrier properties of the assembled membrane, AC (at 48 or 240 nM) was added on the top of the tethered bilayer covering the immobilized CaM (at same density of 45±5 ng/cm^2^). After extensive washing of the AC solution, the bilayer was disrupted by addition of a non-ionic detergent (0.1% Triton X-100), and the enzymatic activity was monitored. As shown in Figure 7, no detectable AC activity was found indicating that the AC molecules added above the tethered bilayer had no access to the underlying CaM polypeptides (Figure 7c). In contrast, when the bilayer was removed by the Triton wash prior to the addition of AC (Figure 7d), a high amount of AC activity was detected demonstrating that the immobilized CaM underlying the membrane was fully functional in AC binding and activation of its enzymatic activity. All together these results indicated that the tethered bilayer assembled over the CaM cushion constituted a truly effective barrier that precluded the passage of AC molecules from the bulk medium to the underlying compartment.

### Conclusion

We have demonstrated here the construction of a multilayered biomimetic assembly that recapitulates the fundamental properties of an authentic biological membrane in creating a tightly sealed, protein-impermeable boundary between two distinct compartments, a *“cis”* side corresponding to the extracellular milieu and a *“trans”* side incorporating a key signalling protein, CaM, as a marker of the cytosolic environment. CaM was immobilized in a fully functional state in the underlying *“trans”* side and was completely insulated from the *“cis”* compartment and inaccessible to a CaM-dependent enzyme, unless the bilayer was disrupted by non-ionic detergents. The simplicity and robustness of the assembly procedure described here should facilitate the elaboration of biomimetic membrane models incorporating not only membrane embedded proteins but also key cytosolic components. This will help in reconstituting multi-molecular membrane-associated machineries including membrane transport systems and/or signalling complexes. Furthermore, the currently designed biomimetic membrane is a promising tool to study the translocation of proteins across membranes.

## Materials and Methods

### Proteins

Calmodulin (CaM) and the catalytic domain of the adenylate cylase toxin (AC, corresponding to the first 384 residues of the CyaA toxin[15]) were produced in the *Escherichia coli* strain BLR (Novagen), transformed with plasmids pDLTCaM41 or pTRAC384GK respectively, and purified to homogeneity as previously described.[19] The protein purity in both preparations was higher than 95% as judged by sodium dodecyl sulfate-polyacrylamide gel electrophoresis (SDS-PAGE). Protein content was determined from absorption spectra using an extinction coefficient at 278 nm of 2,800 M^−1^.cm^−1^ for CaM and 28,000 M^−1^.cm^−1^ for AC. Proteins were stored frozen in 20 mM HEPES (N-(2-hydroxyethyl)-piperazine-N'-2-ethanesulfonic acid)-Na buffer, 150 mM NaCl, 1 mM dithiotreitol, pH 7.4, and diluted into HBS buffer (20 mM Hepes-Na, 150 mM NaCl, 2 mM CaCl_2_, pH 7.4) for all manipulations.

### Preparation of substrates

The glass material used in this work was carefully cleaned with a 2% Hellmanex solution (Hellmanex, France) and thoroughly rinsed with Milli-Q water and then ethanol. We used two different sizes of glass surfaces, depending of the performed measurement. For enzymatic activity measurement, we used round coverslips (Menzer Gläser GmbH, Germany) with a diameter of 14 mm and for surface plasmon resonance (SPR) measurements, we used half glass microscope slides (operating area in the cell: 1.54 cm^2^) (Menzer Gläser GmbH, Germany). Both glass surfaces were coated with 2 nm of chromium and 47 nm of gold by thermal evaporation as previously described. [13] Amino-coated gold surfaces were obtained by overnight self-assembling of 2-mercaptoethylamine (cysteamine hydrochloride, ≥99%, Sigma-Aldrich, 5 mM solution in pure ethanol).

### Calmodulin immobilization onto amino-coated gold surfaces

CaM was covalently coupled to the amine-grafted surface using *N*-(3-dimethylaminopropyl)-*N*'ethylcarbodiimide hydrochloride (EDC) activation, in the presence of 2 mM Ca^2+^. EDC coupling was preferred to EDC/*N*-hydroxysuccinimide (NHS) activation; indeed the shorter half-life of EDC activated carboxylic groups prevented the inter-protein cross-linkages observed with EDC/NHS coupling. For immobilization of CaM on the amino coated-surface, the purified protein at different concentrations (from 1 to 10 µg/mL) in HBS buffer was mixed extemporaneously with a fresh EDC solution (2.5 molar excess over CaM) and 600 µL of this carboxy-activated protein solution were immediately deposited on the amino-coated coverslip as a droplet. Alternatively the carboxy-activated CaM solution was directly injected onto the slides assembled into the SPR measuring cell (see below). After 15 min of reaction at room temperature, the surface was washed with 3 mL of HBS buffer, then washed with 3 mL of a 100 mM ethanolamine (to block the unreacted activated carboxyl) and finally rinsed with 3 mL of HBS buffer. The formation of the protein layer was monitored by SPR spectroscopy.

### Tethered bilayer formation

L-α-Phosphatidyl choline from egg yolk (egg-PC) type XVI-E was purchased from Sigma-Aldrich (St Quentin-Fallavier, France) and 1,2-distearoyl-*sn*-glycero-3-phosphoethanolamine-poly-(ethyleneglycol)-*N*-hydroxysuccinimide (DSPE-PEG_3400_-NHS) was from Shearwater Polymers (Huntsville, AL, US). Vesicles were obtained as described previously.[13] Tethered lipid membrane (*t*BLM) formation was obtained by deposition of mixed Egg-PC/DSPE-PEG_3400_-NHS (95/5 w/w ratio) vesicles in HBS buffer onto the CaM-coated surface. The buffer covering the CaM-coated surface was discarded just before the addition of the vesicle suspension. 600 µL of a mixed vesicle suspension (1 mg/mL) were added directly as a droplet onto the coverslips, or alternatively after mounting the CaM-coated coverslip into the SPR measurement cell. The mixed vesicles were allowed to react for one hour at room temperature, and then the surface was thoroughly rinsed with 3 mL of HBS buffer. In these conditions the bound vesicles spontaneously ruptured and fused to form a continuous lipidic bilayer as described previously. [14]

### SPR measurements

Measurements were performed using a home-made optical set up in Kretschmann configuration as previously described.[13] The amino-gold-coated glass slide was vertically assembled with a PTFE sample cell (600 µL of internal volume) and a 90° high refractive index LaSFN9 prism (n = 1.85) using an immersion oil (Serie A, n = 1.7, Winopal Forschungsbedarf Gmbh, Germany). Monochromatic p-polarized light from a He-Ne laser beam (λ = 633 nm) was reflected from the backside of the gold-coated glass slide. The area of the coated gold surface in contact with the liquid was 1.54 cm^2^. Reflectivity was recorded as a function of the incident angle and the optical thickness was determined according to Fresnel equations using the Winspall program (Max-Planck Institute for Polymer Research, Mainz, Germany).

The amount of lipid on the surface was expressed as the optical thickness (expressed in Å). To determine the amount of protein absorbed onto the surface, optical thicknesses were converted to protein surface density using a conversion factor of 100 ng/cm^2^ for an optical thickness of 10 Å.[14] All measurements were performed at 25°C.

### Fluorescence recovery after photobleaching (FRAP)

For FRAP experiments, the fluorescent probe DPPE-NBD (1,2-dipalmitoyl-*sn*-glycero-3-phosphoethanolamine-*N*-NBD (7-nitro-2,1,3-benzoxadiazol-4-yl), Sigma-Aldrich, St Quentin-Fallavier, France) was added into the vesicles at a 2% molar ratio. The diffusion coefficient and the mobile fraction of the lipids were determined as described previously.[14] The observed areas of the bilayer were 320 * 320 µm^2^ and the photobleaching area was 45 *45 µm^2^.

### Adenylate cyclase (AC) binding assays

For all AC binding assays, the protein was diluted in HBS buffer. Buffer covering the surface (amino coated, CaM-coated or *t*BLM-CaM coated) was very carefully removed before addition of 600 µL AC solution (from 40 to 360 nM) in HBS buffer either deposited as a droplet onto the coverslip, or alternatively by injection over the coverslip assembled into the SPR measuring cell. After 30 min of incubation at 25°C, the AC solution was thoroughly washed with 3 mL of HBS buffer.

The amount of AC bound to the different types of surface was determined from the optical thicknesses using the same conversion factor as above (100 ng/cm^2^ for an optical thickness of 10 Å). Alternatively, the capability of the immobilized CaM to bind and activate AC was determined by measuring the AC enzymatic activity (see below).

In experiments carried out with the *t*BLM assembled over the immobilized CaM surface (*t*BLM-CaM), the tethered bilayer was solubilized, before or after AC binding - depending of the experiments - by washing the surface with 2 mL of 0.1% Triton X-100 (in HBS buffer) and then with 5 mL of HBS buffer.[20] The efficiency of the bilayer removal was checked by SPR spectroscopy and found to be essentially complete in these conditions (data not shown).

### AC enzymatic activity assays

The activity of AC was measured with a sensitive colorimetric assay recently described.[19] AC converts ATP into cAMP and pyrophosphate (PPi). This latter can be further hydrolyzed by an exogenously added pyrophosphatase into two phosphate molecules that can be quantitatively measured with a standard colorimetric assay based on the change in absorbance of the dye malachite green in the presence of phosphomolybdate complexes.

The AC enzymatic assay was carried out in reaction medium composed of 50 mM Tris-HCl, 10 mM MgCl_2_, 0.2% Tween 20, 0.1 mM CaCl_2_, pH = 8.0, supplemented with 2 mM ATP and 2 U/mL of inorganic pyrophosphatase (from *E. coli,* Sigma-Aldrich). The amount of added pyrophosphatase in the reaction medium was checked to be sufficient for an immediate and complete conversion of the released inorganic pyrophosphate into inorganic phosphate (Pi). The amount of Pi produced was determined with the Pi color Lock ^TM^ ALS colorimetric assay from Innova Biosciences (Cambridge, UK).

To measure the AC activity bound to the different types of surfaces, 600 µL of the reaction medium were added to the surface after having thoroughly discarded the washing HBS buffer, and incubated at room temperature. At different period of time, 25 µL samples of the reaction medium were withdrawn and mixed with 100 µL of a Pi ALS mixture (100 µL of Pi color lock +1 µL of accelerator, both provided in the Pi color Lock ^TM^ ALS kit), previously distributed in a well of a 96-wells microplate. The enzymatic reaction was immediately stopped upon dilution of the reaction medium into the Pi ALS mixture due to the acidic pH of this latter.

After 10 minutes of incubation, 10 µL of stabilizer solution (provided in the Pi color Lock ^TM^ ALS kit) were added in each well. After one hour of incubation, the absorbance at 630 nm was recorded using a microplate reader (Dynex, Magellan Biosciences, USA). A calibration curve (not shown) was performed using a Pi standard solution. A linear response (OD_630_ versus Pi concentration) was obtained up to 14 nmoles of Pi (equivalent to 7 nmoles of cAMP or PPi) in the 25 µL sampling volume (data not shown). The initial rates of Pi release were calculated from the linear part of the curves and were converted into initial rate of cAMP formation (v_i_). All v_i_ are given for a reaction medium droplet of 600 µL on a 1.54 cm^2^ surface area.
